# Improved Flow Cytometric Assessment Reveals Distinct Microvesicle (Cell-Derived Microparticle) Signatures in Joint Diseases

**DOI:** 10.1371/journal.pone.0049726

**Published:** 2012-11-20

**Authors:** Bence György, Tamás G. Szabó, Lilla Turiák, Matthew Wright, Petra Herczeg, Zsigmond Lédeczi, Ágnes Kittel, Anna Polgár, Kálmán Tóth, Beáta Dérfalvi, Gergő Zelenák, István Böröcz, Bob Carr, György Nagy, Károly Vékey, Steffen Gay, András Falus, Edit I. Buzás

**Affiliations:** 1 Department of Genetics, Cell- and Immunobiology, Semmelweis University, Budapest, Hungary; 2 Chemical Research Center of the Hungarian Academy of Sciences, Budapest, Hungary; 3 NanoSight Ltd., Amesbury, United Kingdom; 4 Institute of Experimental Medicine, Hungarian Academy of Sciences, Budapest, Hungary; 5 National Institute of Rheumatology and Physiotherapy, Budapest, Hungary; 6 Department of Orthopaedics, University of Szeged, Szeged, Hungary; 7 2^nd^ Department of Pediatrics, Semmelweis University, Budapest, Hungary; 8 Military Hospital, National Health Institute, Department of Orthopaedics, Budapest, Hungary; 9 Department of Rheumatology, Semmelweis University, Budapest, Hungary; 10 Center for Experimental Rheumatology, Zurich Center for Integrative Human Physiology, USZ, Zurich, Switzerland; University of Torino, Italy

## Abstract

**Introduction:**

Microvesicles (MVs), earlier referred to as microparticles, represent a major type of extracellular vesicles currently considered as novel biomarkers in various clinical settings such as autoimmune disorders. However, the analysis of MVs in body fluids has not been fully standardized yet, and there are numerous pitfalls that hinder the correct assessment of these structures.

**Methods:**

In this study, we analyzed synovial fluid (SF) samples of patients with osteoarthritis (OA), rheumatoid arthritis (RA) and juvenile idiopathic arthritis (JIA). To assess factors that may confound MV detection in joint diseases, we used electron microscopy (EM), Nanoparticle Tracking Analysis (NTA) and mass spectrometry (MS). For flow cytometry, a method commonly used for phenotyping and enumeration of MVs, we combined recent advances in the field, and used a novel approach of differential detergent lysis for the exclusion of MV-mimicking non-vesicular signals.

**Results:**

EM and NTA showed that substantial amounts of particles other than MVs were present in SF samples. Beyond known MV-associated proteins, MS analysis also revealed abundant plasma- and immune complex-related proteins in MV preparations. Applying improved flow cytometric analysis, we demonstrate for the first time that CD3^+^ and CD8^+^ T-cell derived SF MVs are highly elevated in patients with RA compared to OA patients (p = 0.027 and p = 0.009, respectively, after Bonferroni corrections). In JIA, we identified reduced numbers of B cell-derived MVs (p = 0.009, after Bonferroni correction).

**Conclusions:**

Our results suggest that improved flow cytometric assessment of MVs facilitates the detection of previously unrecognized disease-associated vesicular signatures.

## Introduction

The release of extracellular vesicles by various types of cells is a ubiquitous process, and accompanies cellular activation and apoptosis [1]. Among the various populations of extracellular vesicles, microvesicles (MVs) (also referred to as microparticles in the literature) appear to be particularly expedient in clinical settings, as they are readily detectable by flow cytometry (FC) in various biological fluids. These vesicles are in between ∼ 80 and 400 nm in diameter [2], [3] in biological fluids, and carry surface molecules that are typical for the cell that releases them. High number of studies suggest that MVs may serve as biomarkers in several diseases including cardiovascular disorders, autoimmune diseases and cancer [1]. However, there are numerous pitfalls that may hinder the correct analysis of MVs especially in biological samples. These include platelet activation in plasma samples induced during collection and transportation of blood samples, residual platelets in platelet-free plasma [4] or sample storage. Furthermore, it has been shown that the isolation procedure itself can have an impact on several parameters of vesicles, and might also induce artificial vesicle release from remaining intact cells or platelets in the ‘platelet-free plasma’ [5]. All these issues may lead to over- or underestimation of platelet-derived MV counts (for a review see [1], [6]). In addition, the presence of immune complexes may also falsify the enumeration of MVs, particularly in diseases associated with accelerated immune complex formation [2]. Novel major improvements have facilitated standard analysis of MVs in biological fluids [7], including improved FC settings and preparation of platelet-free plasma. Recently we have developed a simple method to discriminate between immune complexes and MVs using low concentrations of a detergent by FC [2]. Here we report the results of the first study in which we applied the combination of several improved procedures and recommendations in the MV field for the analysis of synovial fluid (SF) samples.

Excessive formation of SF is a common clinical feature in various arthritides including rheumatoid arthritis (RA), juvenile idiopathic arthritis (JIA), and osteoarthritis (OA). Analysis of SFs in these joint diseases may facilitate the establishment of early diagnosis, and provide an insight into the pathomechanism. Previous studies have shown that SF MVs were detectable by FC [2], and of particular interest, CD41a^+^ vesicles have been shown to be highly elevated in patients with RA compared to OA [9]. However, complex MV signatures have not yet been described.

Using standardized FC settings [7], [8] and applying the novel differential detergent lysis approach (in order to exclude the abundant MV-mimicking non-vesicular events), this is the first study to show various joint disease associated SF MV signatures.

## Materials and Methods

### Patients

Blood plasma was obtained from RA patients (9 women, 3 men, mean age ± s.d.: 60.5±12.0 years; age range: 38–76 years) and from patients with OA (6 women, 3 men, mean age ± s.d.: 53.5±12.79 years; age range: 31–67 years). SF samples from patients with RA (7 women, 1 man, mean age ± s.d.: 50.4±18.5 years; age range 23–73 years, 2 patients were ACPA negative, mean Disease Activity Score (DAS) score was: 5.69), OA (6 women; 2 men, mean age ± s.d.: 64.1±7.9 years; age range 50–71 years) and JIA (6 girls; 4 boys, mean age ± s.d.: 9.5±5.8 years; age range 2–19 years) were included in this study. All RA patients fulfilled the 2010 American College of Rheumatology/European League Against Rheumatism classification criteria [10], [11]. All JIA patients showed oligoarticular manifestations. Patients with RA and OA were treated in the Department of Rheumatology, Semmelweis University (Budapest, Hungary), the National Institute of Rheumatology and Physiotherapy (Budapest) and the Department of Orthopedics, University of Szeged (Szeged, Hungary). Patients with JIA were treated in the 2^nd^ Department of Pediatrics, Semmelweis University (Budapest). Plasma samples drawn into citrate tubes were centrifuged twice at 2,500 g in order to obtain platelet-free blood plasma. SF samples were centrifuged once at 650 g for 20 minutes. Aliquots of the samples were stored at −20°C until use. During the entire investigation period we followed the guidelines and regulations of the Helsinki Declaration in 1975, and the experiments were approved by the Hungarian Scientific and Research Ethics Committee (TUKEB); all patients signed an informed consent form. We obtained an informed written consent from the parents of the children involved in our study. The clinical and serological data of the patients were documented at the time of venipuncture.

### Isolation of Microvesicles

Cell-free SF samples were filtered through an 800 nm filter (Millipore, Billerica, MA, USA) and MVs were pelleted at 20,500 g for 60 minutes in an Eppendorf 5417R centrifuge (Hamburg, Germany), as described previously by many groups [12]–[15]. After resuspending pellets were washed twice with phosphate buffered saline (PBS) and MV preparations were subjected to electron microscopy (EM), nanoparticle tracking analysis (NTA) or mass spectrometry (MS).

### Generation of Immune Complexes

Lactoferrin and anti-lactoferrin and ovalbumin-anti-ovalbumin were mixed at 1∶1 ratio, as described previously [2] (all reagents from Sigma-Aldrich, St. Louis, MO, USA). We also isolated IgM immune complexes from RA SF samples using anti-IgM agarose column, as described previously [2]. Aggregates were analyzed by NTA.

### Electron Microscopy

To visualize MVs, pellets were fixed at RT for 60 minutes with 4% paraformaldehyde. After washing with PBS, the fixed pellets were postfixed in 1% OsO_4_ (Taab, Aldermaston, UK) for 30 minutes, dehydrated in graded ethanol, block stained with 1% uranyl-acetate in 50% ethanol for 30 minutes, and embedded in Taab 812 (Taab). The ultrathin sections were analyzed with a Hitachi 7100 electron microscope (Hitachi, San Jose, CA, USA).

### Nanoparticle Tracking Analysis

For particle size determination, nanoparticle tracking analysis (NTA) was performed with a NanoSight LM10 instrument (NanoSight Ltd., Amesbury, UK) as described previously [16]. NTA captures real-time video files, showing particles moving by Brownian motion. A sample video file recorded by NTA is available in the Supporting Material (Video S1). A 635 nm laser is used to illuminate nanoparticles in liquid suspension between concentrations of 10^6^ and 10^9^ particles per ml. The light scattered from the particles allows them to be visualized using a long working distance ×20 microscope objective fitted to an otherwise conventional microscope. Attached to this microscope is an EMCCD camera running at 30 frames a second which allows a video of the particles moving under Brownian motion to be obtained. The speed of moving particles is determined on a particle-by-particle basis, and the size is calculated using the Stokes-Einstein equation [16]. The concentration of particles is determined directly from the chamber volume and particle number. Immune complexes, native SFs, diluted 1∶1,000 to 1∶10,000 in filtered PBS (depending on particle concentration) as well as isolated MVs were also analyzed using this method. A video of 60 seconds duration was recorded, and data analysis was carried out using the NTA 2.1 analytical software (NanoSight Ltd.).

### Mass Spectrometry

Isolated MVs (see above) were submitted to repeated freeze–thaw cycles and the protein content of the vesicles was digested as reported previously [17]. The LC–MS/(MS) analysis of the tryptic peptide mixtures was performed using a nanoflow ultra performance liquid chromatography system (nanoAcquity UPLC, Waters, Milford, MA, USA) coupled to a Q-TOF Premier mass spectrometer (Waters). All MS/MS samples were analyzed using Mascot (Matrix Science, London, UK; version Mascot 2.2) and X! Tandem (The GPM, thegpm.org; version 2007.01.01.1). X! Tandem was searched against SwissProt_51.6 database assuming trypsin. Mascot was searched against SwissProt_51.6 database assuming digestion enzyme trypsin. One missed cleavage was allowed. Mascot and X! Tandem were searched with a fragment ion mass tolerance of 0.15 Da and a parent ion tolerance of 50 ppm. Iodoacetamide derivative of cysteine was specified in Mascot and X! Tandem as a fixed modification. Scaffold (version Scaffold_3_00_07, Proteome Software Inc., Portland, OR, USA) was used to validate MS/MS based peptide and protein identifications. Peptide identifications were accepted when they could be established at greater than 95.0% probability as specified by the Peptide Prophet algorithm [18]. Protein identifications were accepted when they could be established at greater than 99.0% probability and contained at least 2 identified peptides. Protein probabilities were assigned by the Protein Prophet algorithm [19]. Proteins that contained similar peptides and could not be differentiated based on MS/MS analysis alone were grouped to satisfy the principles of parsimony. Search against the decoy database gave a false-discovery rate of 0.51% when using p<0.01 significance level. Presented results show summarized data from three independent biological replicates, respectively. In case of one sample three technical replicate experiments were also carried out and the results were also taken into consideration.

### Flow Cytometry

Samples were analyzed using a FACSCalibur flow cytometer (BD Biosciences, Franklin Lakes, NJ, USA). The FC instrument settings and MP gating were adopted from previous works [2], [9], [20]. We used a 1 µm bead (Sigma-Aldrich) to determine the upper right corner of the gate [7], (Figure S1a). The lower border was determined after assessing signal/noise ratios and positive event numbers, as described previously [2]. To identify and characterize MVs, annexin V-(AX) fluorescein isothiocyanate, anti-CD3-phycoerythrin, anti-CD4-phycoerythrin, anti-CD8-phycoerythrin, anti-CD14-peridinin chlorophyll protein, anti-CD19-peridinin chlorophyll protein, anti-CD41a-fluorescein isothiocyanate, anti-receptor activator of nuclear factor kappa B (RANK)-phycoerythrin and anti-RANK ligand (RANKL)-phycoerythrin were used (all from BD Biosciences) (Figure S1b). We applied our earlier protocol for staining MVs in biological fluids, with some modifications [2], [21]. Briefly, 1 µg of antibodies was added to 20 µl of plasma/SF, and incubated for 30 minutes at RT. To reduce the numbers of background events, samples were diluted to 400 µl in 0.01 µm pore size membrane-filtered PBS (Millipore). In the case of AX staining, we added 2 µl of AX-fluorescein isothiocyanate to the samples diluted 1∶20 in annexin-binding buffer (BD Biosciences). Event numbers of equal sample volumes were counted for 60 seconds. Background fluorescence was compared with that of the isotype-control antibodies. When detecting AX binding, 5 mM ethylenediaminetetraacetic acid-containing annexin-binding buffer solution was used to determine the background fluorescence. To determine the exact MV concentrations, we used fluorescent counting beads (3 µm in diameter, Partec GmbH, Münster, Germany). We enumerated the counting beads on the SSC/FL-2 plot (Figure S1c). To determine MV concentrations, we used the following equation: ‘MV count/ml = (MV event count/bead event count) × bead concentration × plasma dilution’.

To exclude protein aggregates and immune complex-related events, we applied the method of differential detergent lysis.

### Differential Detergent Lysis

In order to discriminate between MVs and protein aggregates, we benefited from our previous observations that vesicular structures were more susceptible to detergent lysis compared to immune complexes and protein aggregates [2]. By FC, we applied 0.1% of Triton X-100, a concentration which had been determined in preliminary measurements. Events resistant to this concentration were excluded from MV counts, therefore event count *after* Triton X-100 lysis was subtracted the from event number *before* lysis. Remaining event counts were referred to known concentration of fluorescent counting beads. The effect of the differential detergent lysis was also confirmed using a BD LSRII flow cytometer (BD Biosciences).

### Statistical Analysis

SPSS software Version 15.0 was used for statistical analyses. We used Kruskal-Wallis nonparametric test to analyze data of RA, OA and JIA groups. As a post-hoc test, we compared individual groups using the Mann-Whitney test, and we used Bonferroni correction for multiple statistical testing. Correlations between clinical and serologic data were analyzed using Spearman or Pearson correlation.

## Results

### Analysis of the MV Pellet by EM and NTA

**Figure 1:**
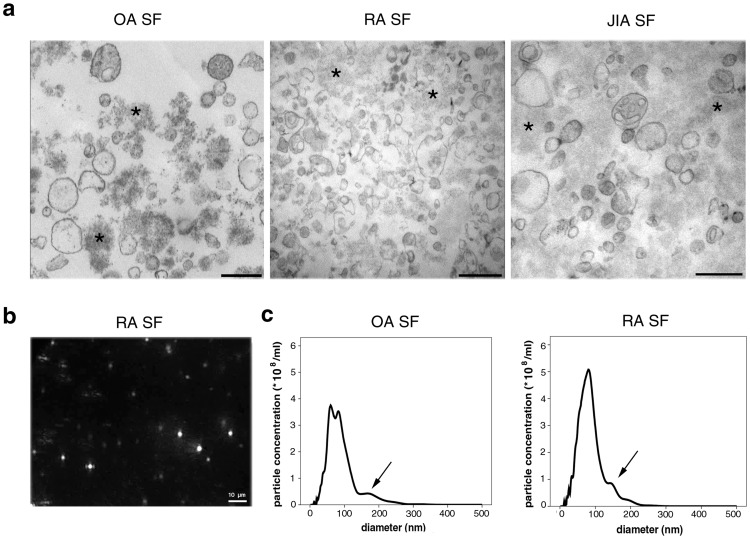
Analysis of SF MVs by EM and NTA. (a). Electron micrographs of the 20,500 g pellets from SFs are shown. Scale bars denote 500 nm, original magnification was 30,000×. (b). NTA screenshot from an RA SF pellet, showing light scattering particles. The field of view is 120 µm by 80 µm. (c). Size histograms of particles in SFs obtained by NTA measurements. A small peak is visible in the MV size range indicated by an arrow (between 100 and 200 nm), however, most particles are below 100 nm in diameter.

EM images of SF-derived MV preparations (20,500 g pellets) clearly showed vesicular structures of 80–400 nm in diameter in all tested SFs (n = 3 in each patient group) (Figure 1a). This size range was consistent with our previous findings [2]. Strikingly, significant amounts of amorphous substance were also present among the vesicles in all EM preparations, in spite of the fact that the pellets were washed twice. MV preparations from RA and OA SFs were subsequently analyzed by NTA, and light scattering of particles was recorded (an example is shown in Video S1). Based on the NTA video files, we determined distributions of particle size in the 20,500 g pellets of RA and OA SF preparations (Figure 1b and 1c). A peak in the MV diameter range (determined by EM, see above) was detectable at around 150–200 nm (Figure 1c). However, strikingly, a significant portion of particles did not fall into the size range of MVs: in the case of OA and RA samples, 60.5% ±12.5% and 50.4% ±6.0% of the particles fell below 80 nm in diameter, respectively. These events are likely to be non-vesicular structures, as MVs smaller than 80 nm were not detected in the EM preparations from the same samples. The application of 0.1% TritonX-100 resulted in the reduction in the particle concentration between 100 and 200 nm and increased the concentration of particles below 100 nm (Figure S2). Next, we analyzed if pure immune complexes and protein aggregates formed particles, detectable by NTA. The size histograms of artificial immune complexes (lactoferrin-anti-lactoferrin, ovalbumin-anti-ovalbumin, isolated IgM immune complexes) and protein aggregates (in BSA solution) partly overlapped the MV size range, but most of the events were smaller than 100 nm in diameter (Figure S3). Taken together these data, these results suggest that particles other than MVs (protein aggregates, immune complexes) are also present in relatively high numbers in the 20,500 g SF pellets.

### MS Analysis of MV Pellets

To further analyze the nature of non MV-related events found in MV preparations of OA, RA and JIA SFs, we performed MS analysis of the 20,500 g pellets (n = 3 in each group). Total protein concentrations in RA pellets (mean ± s.d: 0.88±0.63 mg/ml) and JIA pellets (mean ± s.d: 0.51±0.48 mg/ml) were higher compared to OA samples (mean ± s.d: 0.14±0.10 mg/ml) (not significant). We identified 105, 98 and 88 proteins in OA, RA and in JIA pellets, respectively (Table S1). Among the identified proteins, known MV-associated proteins were clearly detectable in all the samples. These included cytoskeletal components (actin, actin-binding proteins (profilin-1, cofilin-1), myosin, tubulin), enzymes (alpha-enolase, pyruvate kinase, triosephosphate isomerase), membrane molecules (HLA-I, HLA-II antigens, Na/K ATP-ase) and proteins involved in vesicle biogenesis and trafficking (e.g. Ras-related proteins). The presence of lactadherin that binds to the phosphatidyl-serine surface of MVs [22], also confirms the presence of MVs in the pellets. We also detected clusterin (ApoJ) in all patient groups, a protein involved in the clearance of apoptotic bodies and cell debris [23]. Besides canonical MV proteins, numerous plasma proteins (e.g. albumin, transferrin, fibrinogen, prothrombin, haptoglobin) and immune-complex related proteins (complement proteins and immunoglobulins) were also identified by MS. These results further support the hypothesis that MV pellets (prepared by conventional differential centrifugation), contain high numbers of plasma proteins and immune-complex related proteins as non-vesicular contaminants. Most interestingly, the identified protein patterns were almost identical in all samples irrespectively of the type of the joint disease (OA, RA or JIA) (Table S1).

### Flow Cytometric Analysis of Samples

**Figure 2:**
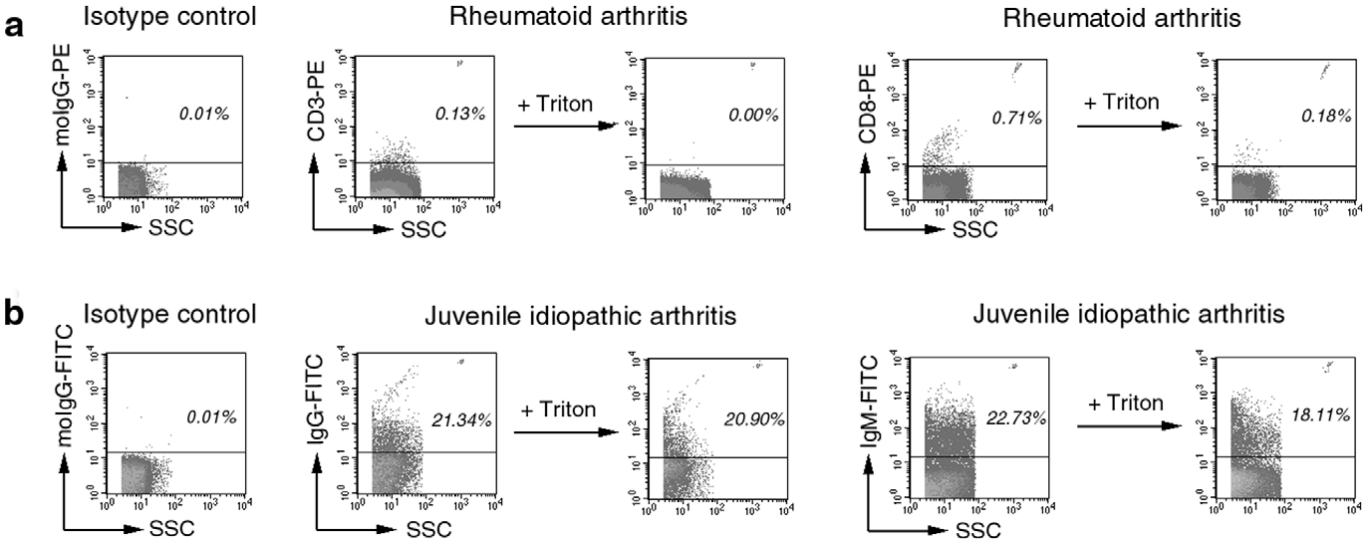
Differential detergent lysis by FC. Events are shown within the MV gate. Background fluorescence was determined using an isotype control antibodies. Values show percents of positive events. Counting beads are visible in the upper right corner of the dot plots. (a). CD3^+^ and CD8^+^ vesicles were detected by FC. Most positive events disappear after the addition of 0.1% Triton X-100. Remaining events were subtracted from the original event counts. (b). Detergent-resistant IgG and IgM staining in JIA SF samples suggested that these events were related to immune complexes rather than vesicular structures.

Routine diagnostic assessment of MVs comprises the analysis of diluted biological samples. Previously we have shown that the presence of immune complexes may falsify the estimation of MV counts [2]. Using differential detergent lysis, we could identify MVs in SF samples (Figure 2a) based on both surface staining and detergent sensitivity. With this approach, immune complex particles were also detected in JIA SF samples (Figure 2b), similarly to what had been shown previously in the case of RA [2]. Interestingly, in the case of certain cell surface labeling, positive event counts were not reduced after detergent lysis. A striking example is shown in Figure S4a where CD68 staining of MVs was carried out by a standard BD antibody. Events within the MV gate did not disappear after the addition of detergent and were also present in PBS and in BSA solutions (Figure S4b). These MV mimicking, anti CD68 fluorescence signals became stronger with increasing concentration of BSA (Figure S4c). As all biological samples have different protein concentrations, aggregation of CD68 antibodies may also be different leading to false conclusions, if detergent lysis is not used.

### Immunophenotyping of MVs by FC

**Figure 3:**
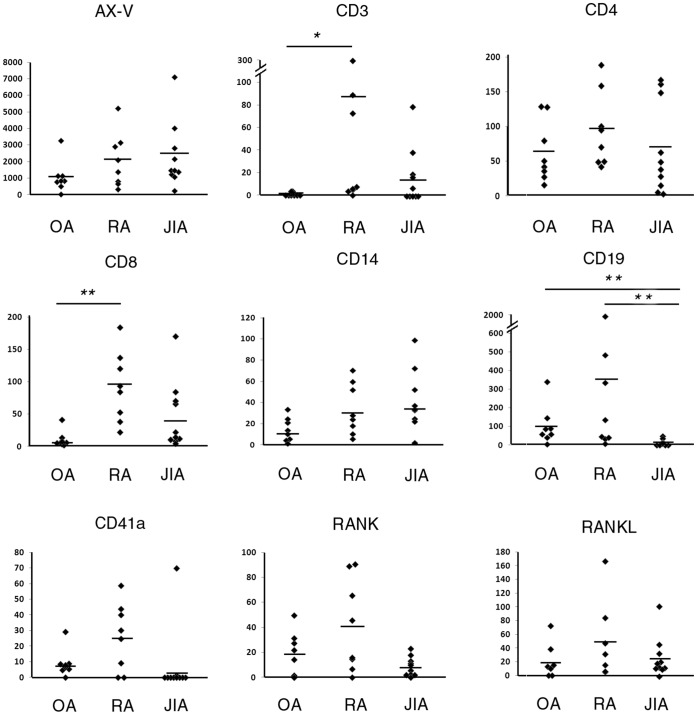
Immunophenotyping of SF MVs by FC. MV count/µl is shown in the y axis. *p<0.05, **p<0.01.

Using differential detergent lysis and counting beads, we phenotyped and counted MVs in RA (n = 8), OA (n = 8) and JIA SF samples (n = 10) (Figure 3). AX^+^ MV counts in SF were slightly, however, not significantly elevated in RA (range:1.0×10^7^–6.5×10^7^/ml) and in JIA patients (range: 4.5×10^6^–1.4×10^8^/ml) compared to OA (range: 6.8×10^6^–1.0×10^8^/ml) consistent with our previous findings [2] (Figure 3). Most vesicles were derived from B-cells and T-cells in the case of OA and RA SF samples. Monocyte- and platelet MVs were present in lower amounts in SFs. Synovial CD41a^+^ vesicles were elevated in RA compared to OA and JIA patients, in concordance with previous findings [9], however, this elevation was not significant. Most interestingly, CD3^+^ T-cell derived vesicles were highly elevated in patients with RA compared to OA (p = 0.027, Mann-Whitney test, Bonferroni correction) in our study.

Next, we tested T-cell subset-derived MVs in SFs. Both CD4^+^ and CD8^+^ vesicles were present in the samples, however, only CD8^+^ MVs showed significant elevation in RA compared to OA (p = 0.009, Mann-Whitney test, Bonferroni correction). Interestingly, B-cell-derived vesicle counts were significantly lower in JIA compared to OA and RA samples (p = 0.009 and p = 0.004, respectively, for B-cells, Mann-Whitney test, after correction). Counts of CD8^+^ and CD3^+^ events showed correlation in the samples (R = 0.525, p = 0.007, Spearman correlation). To assess whether CD3^+^ and CD8^+^ counts of vesicles were elevated not only locally but also systematically in RA patients, we analyzed RA and OA plasma samples. CD3^+^ and CD8^+^ vesicle counts were undetectable in the blood plasma in both groups, indicating the local production of these MVs (p<0.001, Mann-Whitney test) (data not shown).

Given that the tested joint diseases are known to be accompanied by bone erosions, we also tested whether the RANK and RANK-ligand were associated with MVs. Interestingly, we could detect vesicles carrying these surface molecules in all groups, however, very few MVs were stained (Figure 3). The highest numbers were detected in RA; however, the differences among disease groups were not significant.

### Associations with Clinical Parameters

Finally we tested whether the number of MVs correlated with either the clinical or the serological data of patients. Interestingly, the level of rheumatoid factor (RF) showed robust correlations with both T-cell- and B-cell-derived MV numbers in RA (Pearson correlation, R = 0.912, p = 0.002 and R = 0.956, p = 0.001, respectively). T- and B-cell-derived MV counts also showed strong correlation with one another (p<0.001, R = 0.876). DAS Score, Visual Analogue Scale score and anti-citrullinated protein antibody levels did not correlate with MV counts. However, disease duration positively correlated with the CD41a^+^ MV counts (Pearson correlation, R = 0.886, p = 0.008). SF cell number showed only a weak correlation with CD3^+^ and CD8^+^ MV counts (Pearson correlation, R = 0.883, p = 0.039 and R = 0.892, p = 0.017), and showed no correlation with other MV phenotypes, suggesting that the count of MVs reflect the state of cellular activation rather than the number of the cells.

## Discussion

In the past few years, the number of publications assessing MVs by flow cytometry platforms has risen rapidly. However, pitfalls of MV measurement have also received increasing attention in the past few years. Efforts to standardize sample collection and FC measurements have already led to numerous results; however, the standardization work is still in progress [7], [8]. In our present study we aimed at benefiting from recent developments in this field in order to reveal novel disease-associated MV signatures. These developments included i) the use of standard preanalytical conditions, ii) bead-based definition of MV gates, iii) bead-based MV enumeration, and iv) discrimination of MVs from non-vesicular structures.

Biological fluids contain high amounts of proteins, which may form aggregates or complexes under certain circumstances. We have shown earlier that protein aggregates share biophysical parameters with MVs that may confound correct assessment of these structures [2]. Using EM and NTA, here we demonstrated the presence and abundance of particles other than vesicular structures in MV pellets. These non-vesicular structures may correspond to immune-complexes, protein aggregates [2] or membrane fragments formed during centrifugation. In addition, they might derive from lysed, disrupted MVs [5]. Centrifugation alone is not sufficient to pellet out all cells, for instance even after a 2,880 g 20 minutes and a subsequent 10,840 g 5 minutes centrifugation, platelets have been shown to be still present in the ‘platelet-free plasma’ (up to a concentration of 47/µl, which corresponded to 0.02% of the original platelet count) [4]. The high-speed centrifugation of cells during MV isolation results in high shear stress that may tear off membrane fragments or even vesicles from these remaining cells [5]. However, fragmentation of cells during the 20,500 g centrifugation probably did not contribute to the amorphous structures seen by EM in our study, because we used a gentle, gravity driven filtration of the SFs through an 800 nm membrane prior to centrifugation. To our surprise, using NTA we detected predominantly very small sized particles in 20,500 g pellets. EM analyses of numerous samples, however, ruled out the possibility that small size (<80 nm) vesicles (easily recognized based on membrane osmium staining) were present in these preparations. Therefore, the abundant light scattering small ‘particles’ in NTA were more likely protein aggregates rather than vesicles. MS analysis in this work also demonstrated the presence of primarily non-MV related proteins. Although it can not be excluded that SF MVs can harbor certain plasma proteins, the presence and abundance of nearly all major plasma- and immune complex-related proteins in the MV preparations makes it unlikely that all these proteins were associated with vesicles.

Improved MV measurement by FC should exclude these non-MV related events. For discrimination, we suggested the use of differential detergent lysis method. In this paper we provided an example how omission of this step may lead to false conclusions, even when using standard, controlled antibodies.

Interestingly, total particle counts, measured by NTA, were two orders of magnitude higher than the total (AX^+^) counts detected by FC. This supports the ‘iceberg’ theory which assumes that FC only detects particles above 200–300 nm (although the detection threshold is also dependent on the refractive index of the particles, [24]) and most of the particles in SFs fall below this range. On the other hand, NTA detects any particles, whereas by FC we enumerated only the true (AX-positive, Triton sensitive) vesicle-related events. However, using the fluorescence capability of the NTA system and specific labeling, individual populations may also be analyzed in the future [16].

After using the differential detergent lysis approach to evaluate MV signatures in SFs, we observed for the first time that CD3^+^ and CD8^+^ MVs were highly elevated in patients with RA compared to OA.

T-cells, in particular CD4^+^ cells have been implicated in the pathogenesis of RA as summarized by a recent review [25]. In spite of numerous observations that suggested a role of CD8^+^ T cells in RA, CD8^+^ T cell involvement is absent from the current mainstream understanding of the pathomechanism of RA. Evidence for the role of CD8+ T cells includes that CD8^+^ T cells were present in high numbers in the SFs of RA patients [26], [27]. Moreover, phenotype analysis of SF T-cells confirmed that the accumulating T-cell clones in RA were predominantly CD8^+^ memory T cells [26]. Furthermore, memory CD8^+^ T-cells in RA were found to be specific to viruses, such as Epstein-Barr virus, cytomegalovirus or influenza virus [28]. It has also been suggested that CD8^+^ cells may contribute to the pathogenesis of RA by regulating structural integrity and functional activity of germinal centers in the synovium [29]. The MV signatures that we detected in this study may reflect either elevated SF CD8^+^ T cell numbers or CD8^+^ T cell activation in the RA joints. The latter may have importance, since CD8^+^ and CD4^+^ T cell counts were found to be similar in both RA and OA [30]. Thus, our data might reflect a locally enhanced activation of the CD8^+^ T cell population. As part of the SF MV signature of RA, here we also found an elevated number of platelet-derived in RA SFs in accordance with the findings of Boilard et al [9]. Similarly, the number of both RANK and RANKL positive MVs was found to be elevated in RA SFs.

Even though JIA is an inflammatory disease characterized by the proliferation of B-cells, in this study we detected a strikingly reduced number of B-cell-derived MVs in SFs of patients with JIA. This was possibly due to the fact that we analyzed only JIA patients with oligoarticular involvement. As shown in a recent report, synovial CD20 expression differs significantly between polyarticular and oligoarticular JIA patient groups [31]. Patients with polyarticular involvement have three times higher expression of CD20 in the synovium. Our present study suggests that oligoarticular JIA may have distinct MV signature from other joint diseases.

In conclusion, our data suggest that the differential detergent lysis method and other improvements in MV assessment by FC may provide important novel insights into the pathomechanism of RA and other joint diseases.

## Supporting Information

Figure S1 MV gate (R1) was determined using a 1 µm calibration bead (arrow) (a) Arrowhead shows counting beads (3 µm in diameter). MVs were stained with AX, events are shown from the MV gate (b). The background was determined adding 5 mM EDTA to the samples. The number of counting beads was determined on the SSC/FL-2 dot plot (R2 gate) (c).
(TIF)

Figure S2 Size histograms of particles in an MV preparation from an RA patient, obtained by NTA. Dashed line represents 0.1% TritonX-100 treated sample.
(TIF)

Figure S3 Size histograms of immune complexes and protein aggregates measured by NTA. Lactoferrin (LF) was mixed 1∶1 with anti-lactoferrin, ovalbumin (OVA) was mixed 1∶1 with anti-ovalbumin. IgM immune complexes were isolated from RA SF using anti-IgM agarose column.
(TIF)

Figure S4 Flow cytometric MV-mimicking signals using an anti-CD68 antibody. The fluorescent events do not disappear after detergent lysis in blood plasma (a) and in BSA solution (b). The increasing concentration of BSA results in stronger fluorescent signals. The y axis represents event number/µl (c).
(TIF)

Table S1 MS analysis of MV preparations from patients with RA, OA and JIA.
(XLS)

Video S1 Sample NTA screenshot of an MV preparation from an RA patient. The field of view is 120 µm by 80 µm.
(WMV)
