# Supplementary figures and images for: Improved Flow Cytometric Assessment Reveals Distinct Microvesicle (Cell-Derived Microparticle) Signatures in Joint Diseases
Source: PLoS One. 2012 Nov 20;7(11):e49726. doi: 10.1371/journal.pone.0049726 (PMC3502255; doi:10.1371/journal.pone.0049726)

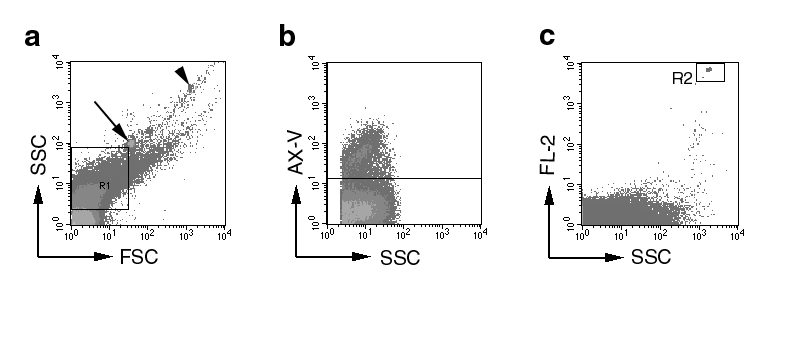

Supplement: Figure S1 — MV gate (R1) was determined using a 1 µm calibration bead (arrow) (a) Arrowhead shows counting beads (3 µm in diameter). MVs were stained with AX, events are shown from the MV gate (b). The background was determined adding 5 mM EDTA to the samples. The number of counting beads was determined on the SSC/FL-2 dot plot (R2 gate) (c). (TIF) [file pone.0049726.s001.tif]

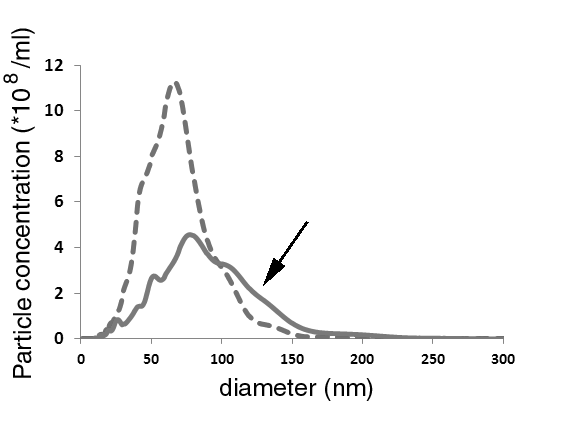

Supplement: Figure S2 — Size histograms of particles in an MV preparation from an RA patient, obtained by NTA. Dashed line represents 0.1% TritonX-100 treated sample. (TIF) [file pone.0049726.s002.tif]

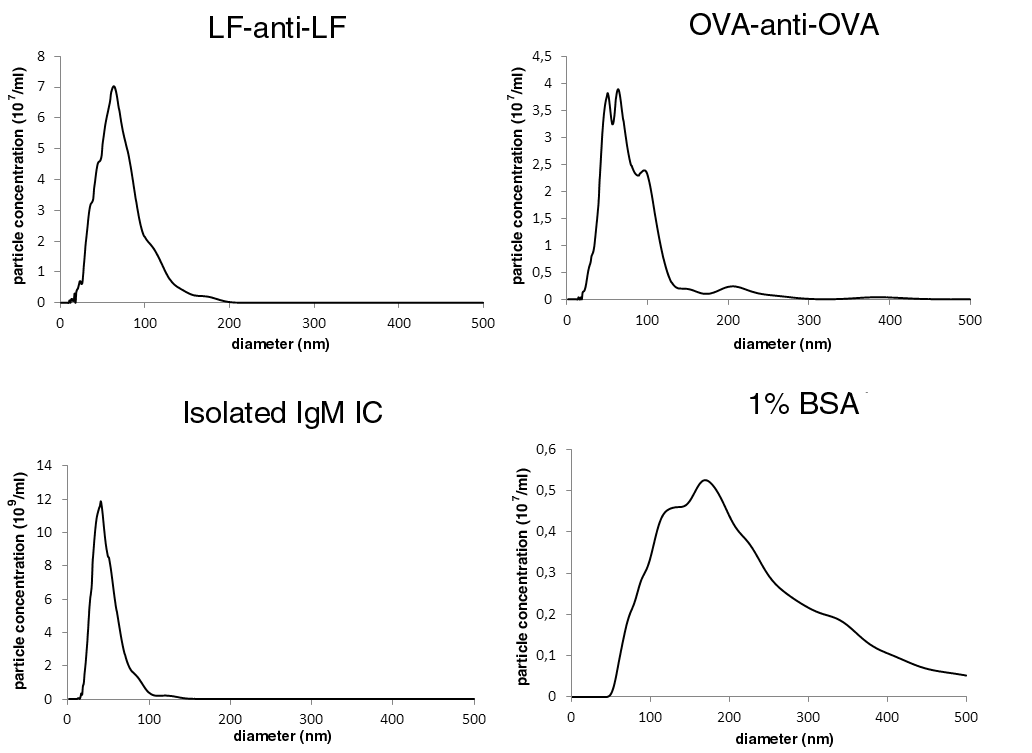

Supplement: Figure S3 — Size histograms of immune complexes and protein aggregates measured by NTA. Lactoferrin (LF) was mixed 1∶1 with anti-lactoferrin, ovalbumin (OVA) was mixed 1∶1 with anti-ovalbumin. IgM immune complexes were isolated from RA SF using anti-IgM agarose column. (TIF) [file pone.0049726.s003.tif]

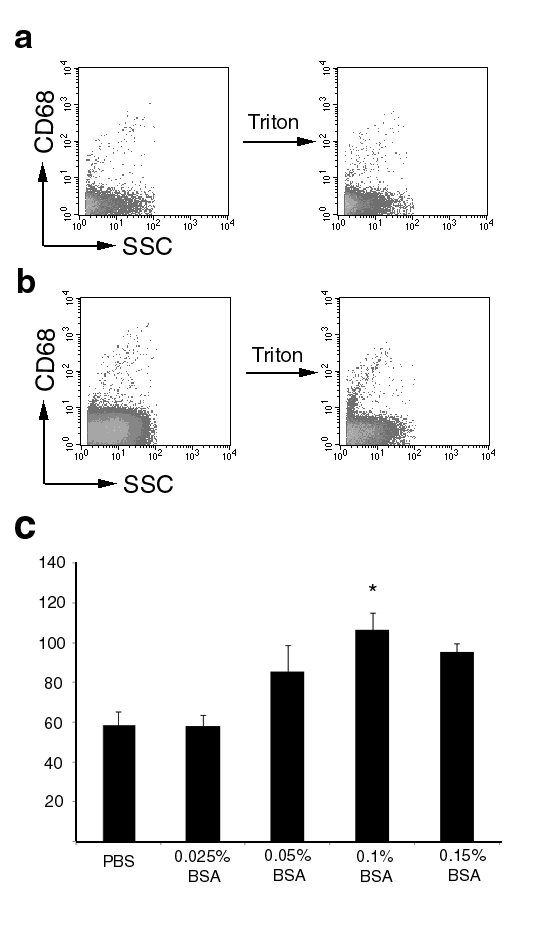

Supplement: Figure S4 — Flow cytometric MV-mimicking signals using an anti-CD68 antibody. The fluorescent events do not disappear after detergent lysis in blood plasma (a) and in BSA solution (b). The increasing concentration of BSA results in stronger fluorescent signals. The y axis represents event number/µl (c). (TIF) [file pone.0049726.s004.tif]
